# UXT is required for spermatogenesis in mice

**DOI:** 10.1371/journal.pone.0195747

**Published:** 2018-04-12

**Authors:** Eric D. Schafler, Phillip A. Thomas, Susan Ha, Yu Wang, Keria Bermudez-Hernandez, Zuojian Tang, David Fenyö, Margarita Vigodner, Susan K. Logan

**Affiliations:** 1 Department of Biochemistry and Molecular Pharmacology, New York University School of Medicine, New York, NY, United States of America; 2 Pathobiology and Translational Medicine Training Program, New York University School of Medicine, New York, NY, United States of America; 3 Department of Urology, New York University School of Medicine, New York, NY, United States of America; 4 Department of Microbiology, New York University School of Medicine, New York, NY, United States of America; 5 Institute for Systems Genetics, New York University Langone Medical Center, New York, New York, United States of America; 6 Center for Health Informatics and Bioinformatics, New York University School of Medicine, New York, NY, United States of America; 7 Department of Biology, Stern College, Yeshiva University, New York, NY, United States of America; 8 Department of Developmental and Molecular Biology, Albert Einstein College of Medicine, Bronx, NY, United States of America; National Cancer Institute, UNITED STATES

## Abstract

Male mammals must simultaneously produce prodigious numbers of sperm and maintain an adequate reserve of stem cells to ensure continuous production of gametes throughout life. Failures in the mechanisms responsible for balancing germ cell differentiation and spermatogonial stem cell (SSC) self-renewal can result in infertility. We discovered a novel requirement for Ubiquitous Expressed Transcript (UXT) in spermatogenesis by developing the first knockout mouse model for this gene. Constitutive deletion of *Uxt* is embryonic lethal, while conditional knockout in the male germline results in a Sertoli cell-only phenotype during the first wave of spermatogenesis that does not recover in the adult. This phenotype begins to manifest between 6 and 7 days post-partum, just before meiotic entry. Gene expression analysis revealed that *Uxt* deletion downregulates the transcription of genes governing SSC self-renewal, differentiation, and meiosis, consistent with its previously defined role as a transcriptional co-factor. Our study has revealed the first *in vivo* function for UXT in the mammalian germline as a regulator of distinct transcriptional programs in SSCs and differentiating spermatogonia.

## Introduction

Spermatogenesis is a complex process of transformation that requires perfect reliability to ensure the accurate transmission of genetic material to the next generation. Failure at any point in this process can compromise an individual’s ability to reproduce. One of the major challenges for male reproduction is to balance the prodigious production of sperm with the maintenance of an adequate stem cell reserve that ensures continuous output throughout life.

According to current models, it is hypothesized that the establishment of the male germ line begins with neonatal gonocytes. Gonocytes can either differentiate into spermatogonial stem cells (SSCs), which self-renew [1], or differentiate into A_1_ spermatogonia, which ultimately become sperm [1, 2]. Gonocytes transitioning into spermatogonial stem cells express the basic helix-loop-helix transcription factor NEUROG3 (NGN3), while gonocytes committed to differentiation will express C-KIT as A_1_ spermatogonia, skipping the SSC and NGN3-positive stages [1, 3]. While this finding has been instrumental in understanding the establishment of the male germline, little is known about the factors that regulate the balance between gonocytes transitioning to form C-KIT positive A_1_ spermatogonia, versus establishing the SSC pool.

A_single_ (A_s_) cells are thought to constitute the SSC population in the adult testis [4], though recent studies suggest that there is heterogeneity within this population with regard to gene expression and stem-like potential [5]. A_s_ first divide to form A_paired_ (A_pr_) spermatogonia which in turn divide to form chains of A_aligned_ (A_al_) spermatogonia, connected by intercellular bridges [6]. A_s_, A_pr_, and A_al_ spermatogonia are collectively called A_undiff_ spermatogonia which can be identified by various markers such as ID4, GFRA1, and NGN3. Other proteins that play a role in maintaining the SSC pool include LIN28A [7, 8], ZBTB16 (PLZF) [9, 10], and DNMT3L [11].

The transition between A_al_ spermatogonia into differentiated type A_1_ spermatogonia is regulated by retinoic acid (RA) which is required for germ cell differentiation [12]. Exposure of adult A_al_ spermatogonia to RA induces the expression of various differentiation markers such as C-KIT and *Stimulated by retinoic acid gene 8* (STRA8), which mark the beginning of meiosis in spermatogenesis [13]. While these findings have been integral to our knowledge surrounding the various signals that are important for differentiation versus self-renewal, the factors that govern the balance between these two processes have yet to be elucidated.

It has been demonstrated that the UXT (also known as ART-27, STAP1, and SKP2 Associated-Alpha PFD1, NM_013840.3) binding partner unconventional prefoldin RPB5 interactor (URI) plays an integral role in germ cell survival, and loss of URI ultimately leads to sterility in *C*. *elegans* [14, 15]. We have further characterized the transcriptional functions of URI, and determined that its stability is highly dependent upon the expression of its binding partner UXT [16]. *UXT* is an X-linked gene that is expressed in a number of somatic human tissues [17]. UXT serves as a co-factor for multiple transcription factors (TFs), including GATA4, NF-κB, androgen receptor (AR), and other nuclear receptors [18–22]. It has been shown that as a co-factor, UXT can possess inhibitor functions in certain growth and development pathways, such as seen with AR, and EVI1 [20, 23]. UXT, in concert with the RPB5 binding partner URI, has also been found to regulate AR target gene expression by binding to gene promoters and enhancers [16, 20].

Despite the interaction of UXT with URI and the importance of URI in germ cells, little is known about the role of UXT in germ cell establishment and maintenance [14, 15]. To assess the role of UXT *in vivo*, we engineered mice carrying a floxed *Uxt*. Constitutive deletion of *Uxt* resulted in embryonic lethality suggesting a critical role for this protein in embryonic development. To address the role of UXT in germ cells, in this report we describe the first knockout mouse model of UXT, which upon specific inactivation of *Uxt* in the male germline exhibited sterility. We demonstrate that the loss of UXT resulted in a dramatic loss of germ cells in a narrow time window during the first spermatogenic wave eventually leading to a Sertoli cell-only phenotype that did not recover in the adult. RNA-seq data together with qPCR analysis and immunohistochemical studies revealed downregulation of important genes responsible for germ cell differentiation and entrance into meiosis, as well as those responsible for establishment and maintenance of SSCs. This work highlights the previously unexplored role of UXT in spermatogenesis, and demonstrates its requirement in germ cell survival and its potential role in the regulation of specific transcriptional programs governing male germ cell differentiation and SSC maintenance.

## Materials and methods

### Generation of *Uxt* conditional knockout mice

*Uxt* ^*F/F*^ mice on a 129/SvJ background were generated by Ozgene (Australia). Genomic fragments approximately 7kb in length were amplified by PCR from C57 Bl/6 genomic DNA to generate 5’ and 3’ homology arms, which included exons 1–2 and exons 4–7, respectively. Exon 3 was amplified separately by PCR using primers containing a 3’ loxP site. The exon 3-loxP arm was then cloned into a target vector downstream of a loxP-FRT-PGK-Neo^R^-FRT selection cassette. The 5’ and 3’ homology arms were subsequently cloned into the target vector, flanking the cassette and exon 3. Forward and reverse sequencing and restriction digestion confirmed the final targeting construct. Homologous recombination of the targeting vector was carried out in W9.5 ES cells derived from 129/SvJ mice. Clones were selected for neomycin resistance, and DNA from resistant clones was used to test genotyping probes. Correctly targeted W9.5 ES clones were identified by Southern blot analysis and microinjected into C57 Bl/6 blastocysts to generate chimeric animals. After establishment of germline transmission, the FRT-PGK-Neo^R^-FRT cassette was deleted by mating to a transgenic line containing FLP recombinase (Oz-Flp). Oz-Flp was removed by mating *Uxt* ^*floxΔNeo/+*^; Oz-Flp mice to wild type C57 Bl/6 mice, resulting in *Uxt* ^*F/+*^ mice of mixed 129/SvJ and C57 Bl/6 background. *Uxt* ^*KO/+*^ mice were generated by mating *Uxt* ^*floxΔNeo/+*^ mice to transgenic mice containing Cre recombinase driven by the Rosa26 promoter (Oz-Cre) of C57 Bl/6 background. These F_1_ generation heterozygous females were then mated to *Uxt* ^*+/Y*^ males to ascertain the Mendelian inheritance pattern of the knockout allele. Mixed background homozygous *Uxt* ^*F/F*^ females were mated to Vasa-Cre (FVB-Tg(Ddx4-cre)1Dcas/J) males (Jackson Laboratory #006954) to generate germ cell-specific conditional knockouts. This study protocol was approved by the Institutional Animal Care and Use Committee of NYU School of Medicine (Protocol 170102). Mice were euthanized with carbon dioxide delivered at a regulated flow rate followed by cervical dislocation. Mice were maintained and observed for signs of distress in accordance with the Guidelines for the Care and Use of Laboratory Animals at NYU School of Medicine.

### Immunohistochemistry

Testes were dissected and fixed in 4% buffered formalin, nutating overnight at room temperature. After fixation, tissues were dehydrated in 50%, 70%, 95%, and 100% ethanol for three changes of 20 minutes each, followed by clearing with two 10 minute changes of xylenes, and overnight paraffin infiltration at 60°C.

Antibody against UXT was generated against the N-terminus of mouse UXT (Covance) and tested for specificity (S1D Fig). For UXT (1:1000), TRA98 (1:200, Abcam ab82527), STRA8 (1:400, Abcam ab49405), PLZF (1:25, Santa Cruz Biotechnology sc-28319), cleaved caspase-3 (1:400, Cell Signaling Technology #9964), GATA-1 (1:100, Cell Signaling Technology #3535), and GATA-4 (1:400, Santa Cruz Biotechnology sc-5093), 5μm tissue sections were deparaffinized in xylenes twice for 10 minutes, then rehydrated twice in 100% ethanol for 5 minutes each, twice in 95% ethanol for 5 minutes each, then running tap water for 10 minutes [24]. Heat-induced antigen retrieval was performed in citrate buffer (8mM sodium citrate, 1.8mM citric acid, pH 6.0) for 20 minutes in a 1200-Watt microwave at 90% power. Slides were cooled then rinsed in PBS. Endogenous peroxidase was quenched with hydrogen peroxide. Sections were blocked with 20% normal goat serum (Vector Laboratories) in PBS for 30 minutes at room temperature in a humid chamber, then incubated with primary antibody at the indicated dilutions in 2% Bovine Serum Albumin (BSA) diluted in PBS overnight at 4°C in a humid chamber. Primary antibody was detected using a biotinylated secondary antibody incubated for 30 minutes at room temperature, followed by R.T.U horseradish-peroxidase streptavidin (Vector Laboratories) for 30 minutes at room temperature, and visualized with the DAB peroxidase substrate kit (Vector Laboratories). Sections were counterstained with Gill’s Formula Hematoxylin (Vector Laboratories H-3401) and mounted with Clear-Mount with Tris Buffer (Electron Microscopy Services) followed by Permount (Fisher), and coverslipped.

### Microscopy

Images for immunohistochemistry were taken on a Zeiss Axio Imager.A2 microscope using a Retiga Exi Camera (Q Imaging) and QCapture Pro 6.0 software. Immunofluoresence images were taken on an EVOS Auto FL Microscope (Life Technologies) at 20x magnification. All immunofluorescence images were taken at the same intensity and exposure across all biological replicates.

### Immunofluorescence

Slides were treated as described above for immunohistochemistry with the following differences. After antigen retrieval with citrate buffer, sections were incubated in 100mM glycine in PBS for 30 minutes at room temperature to quench reactive aldehydes from fixation, blocked in serum, and incubated overnight with both KI-67 (Clone SP6) (1:200, Thermo Scientific #RM-9106) and TRA98 (1:50, Abcam ab82527) primary antibodies at 4°C. Slides were then simultaneously incubated with goat anti-rabbit Alexa Fluor 594 (H+L) and goat anti-rat Alexa Fluor 647 (H+L) secondary antibodies at a 1:5000 dilution in 2% BSA for 30 minutes at room temperature. After washing in PBS, nuclei were stained with 1μg/ml DAPI for 5 minutes. Slides were then mounted with Fluoromount-G (SouthernBiotech) and then coverslipped. Negative controls were treated identically, except sections were incubated in 2% BSA without primary antibody at that step.

### Quantification of immunohistochemistry images

To quantify TRA98, PLZF, C-KIT, and STRA8-positive staining, the number of positively stained cells per tubule was averaged from ≥40 circular tubule cross-sections from non-consecutive testes tissue sections per mouse. Unpaired Student’s t tests or chi square tests were performed on averages from n mice. To quantify cleaved caspase-3-positive staining, we counted the number of tubules containing at least one positive cell across >50 tubules for each biological replicate; an average of 107 WT and 113KO tubules were counted. To conserve tissue, we took into account oblique tubule cross-sections. To do this, we calculated the diameter of one circular tubule and measured this diameter across the oblique section. One circular tubule diameter length was counted as one tubule. We took a random sample of oblique and circular tubule cross-sections across all tissue sections, and assumed that all testes sections contained equal distributions of oblique and circular tubules. In the above quantification the intensity of positive staining was not quantified, only the presence or absence of signal.

### Machine learning for quantification of immunofluorescence images

The automated processing of the images of TRA98 and KI-67 was implemented in Python in the following manner. Segmentation of germ cell nuclei was achieved by using fluorescent images of *Uxt* ^*F/Y*^ and *Uxt* ^*F/Y*^; Vasa-Cre testis tissue sections stained for TRA98 (green). First, the Otsu algorithm [25] was applied to the TRA98 gray-scale image to determine a threshold value to generate a binary image. After segmentation, nuclei found at the edges of the image were eliminated from further analysis and the watershed algorithm was used to separate touching nuclei.

The red channel was then obtained from KI-67 immunofluorescent images. Since KI-67 also labels somatic cell types, the features of the KI-67 image were extracted only from the area covered by the TRA98-positive nucleus. For each cell nucleus the following measures were obtained: maximum red intensity value, sum of red intensity values, mean red intensity, area, and length of major axis. The percentage of nuclear area covered by KI-67 was calculated by first applying the Otsu algorithm [25] to the KI-67 fluorescent image. Then, for each nucleus, the percentage of nuclear area was calculated by measuring the area of red above the threshold and dividing it by the area of the nucleus.

We used a logistic regression classifier to identify KI-67(+) and KI-67(-) nuclei based on these measures [26]. A total of 226 randomly chosen nuclei from three sections that belonged to three separate 6dpp *Uxt* ^*F/Y*^ biological replicates were manually classified by an experimenter to train and evaluate the performance of the algorithm. 127 cells were manually classified as KI-67(+) and 69 were manually classified as KI-67(-). To train the algorithm, the manually classified cells were divided into three sets: a training set of 96 cells (48 KI-67(+), 48 KI-67(-)); a testing set of 42 cells (21 KI-67(+), 21 KI-67(-)); and a testing set of (88 KI-67(+)). To train the logistic regression classifier, a combination of the nuclei measurements was used.

The performance of the algorithm was evaluated by calculating the accuracy. For instance, the algorithm misclassified 5 out of 42 in the first testing set and 14 out of 88 in the second testing set, resulting in an accuracy of 0.88 and 0.84 respectively. The classification algorithm was applied to images obtained from at least three non-overlapping fields from one tissue section at 20x magnification, counting between 400 and 2600 total nuclei per biological replicate. The following python packages were used for the analysis: OpenCV [27], mahotas [28], scikit-image [29], and scikit learn [26].

### RNA isolation

Testes were dissected by removing the tunica, snap-frozen in a dry ice-ethanol bath, and stored at -80°C until RNA isolation. RNA was isolated from one testis each, from three pairs of mixed background, 6-day-old mice from different mating cages. Pairs included one *Uxt* ^*F/Y*^; Vasa-Cre and one *Uxt* ^*F/Y*^ littermate control. All six whole testes samples were disrupted on the same day using a 1.5ml plastic pestle (Kimble Chase) in a 1.5ml Eppendorf tube containing 300μl Buffer RLT Plus (Qiagen) with 1% 2-mercaptoethanol, followed by rotor-stator disruption. To homogenize, the lysate was spun through a Qiashredder column (Qiagen). RNA was purified using the RNeasy Plus Micro kit (Qiagen) with gDNA removal column according to the manufacturer's instructions. After isolating RNA in 14μl water, any residual gDNA contamination was removed using the Ambion TURBO DNA-free Kit in a 50μL reaction volume. Purity, concentration, and RNA integrity were assessed using a Nanodrop (Thermo Fisher Scientific) and the Agilent Bioanalyzer 2100.

### Library preparation and RNA-seq

RNASeq libraries were prepared from 500ng total RNA using the Illumina TruSeq polyA v2 kit following the manufacturer’s protocol, with the exception that 13 cycles of PCR were performed to amplify the libraries to keep the duplication rate lower than with the recommended 15 cycles. The amplified libraries were purified using AMPure beads, quantified by Qubit and qPCR, and visualized in an Agilent Bioanalyzer. The libraries were normalized to 4nM, pooled in an equimolar fashion, distributed uniformly across 2 lanes on high output HiSeq 2500 flow cells using v4 reagents, and sequenced as single-end, 50-nucleotide reads. Library preparation, quality control, and sequencing were performed by the NYULMC Genome Technology Center.

### RNA-seq analysis

Sequence files were generated in FASTQ format by Bcl2fastq version 1.8.4 [30]. Greater than 95% of all base pairs in all samples had a Q score of 30, which corresponds to a calling accuracy of 99.9%. 76.6 million to 95.9 million qualified, 50bp single-end reads were generated in total. Alignments to mouse genome version mm9 were done by TopHat version 2.0.9 [31] with—segment-length 25 -g 1—library-type fr-unstranded -x 1—fusion-search settings. Overall alignment rate ranged between 91% and 96%. Gene count was done by HTSeq [32] version 0.6.1 with—minaqual = 10—mode = union settings. R package edgeR version 3.4.2 [33] was used to find differentially expressed genes. Low expressed genes have been removed in order to separate groups well. We kept genes that achieved at least one base pair per million (cpm) mapped base pairs in at least three replicates. RNA-seq data files are available on NCBI’s Gene Expression Omnibus: accession number GSE111740.

## Results

### *Uxt* deletion results in embryonic lethality

To assess the role of UXT *in vivo*, we engineered mice carrying a floxed *Uxt* allele through homologous recombination in embryonic stem cells. The first exon of *Uxt* is non-coding and the loxP sites flank exon 3, leaving the the original transcription start site intact in exon 2. Excision of exon 3 by Cre recombinase driven by the Rosa26 promoter results in a 1-base pair frameshift mutation in exon 4 that generates a new STOP codon in exon 5 (S1A Fig). This truncates the wild type protein resulting in a short peptide that is likely to be unstable and that does not contain any portion of the prefoldin-like structural domain [19, 34]. While such a strategy may result in generation of a short peptide, this peptide is likely to be unstable and represents less of a risk than deleting the ATG, which could disrupt the promoter and increase the chance that an alternative start site could be used to drive expression of part of the gene. Southern blotting was used to confirm the presence of the floxed allele (labeled *Uxt*^*F*^*)* (S1B Fig). We also developed a PCR genotyping assay using primers recognizing all three alleles (S1C Fig).

Although *Uxt* ^*KO/*+^ females are fertile, we could not generate hemizygous *Uxt* ^*KO/Y*^ male or *Uxt* ^*KO/*+^ female progeny by crossing heterozygous *Uxt* ^*KO/*+^ mothers to wild type males (Table 1). This is likely related to the fact that in mice, the paternal X chromosome is inactivated in extra-embryonic tissues, whereas the epiblast which gives rise to the fetus, is mosaic [35]. Therefore, we hypothesized that *Uxt* deletion would cause a defect in extra-embryonic tissues, since an inactivated, paternal wild type X chromosome cannot rescue a maternal knockout allele in female fetuses. To test this, we set up the reverse cross, mating *Uxt* ^*F/Y*^ males to wild type females expressing Oz-Cre, which is active in early embryogenesis. In female fetuses, we expected the maternal wild type allele to rescue the paternal *Uxt* ^*KO*^ allele, rescuing the knockout in extra-embryonic tissues. Indeed, we were able to generate *Uxt* ^*KO/+*^ female progeny, though no male hemizygotes were observed (Table 2). These data suggest that UXT is required for development of extra-embryonic tissues.

**Table 1:** *Uxt*^*KO/Y*^ male or *Uxt*^*KO/*+^ results in embryonic lethality.

	*Uxt*^*KO/+*^ x *Uxt*^*+/Y*^	
	Observed	Expected
Male WT	20	15.5
Male KO	0	15.5
Female WT	42	15.5
Female KO	0	15.5
	χ^2^: p = 1.92e-10	

**Table 2:** UXT is required for development of extra-embryonic tissues.

	*Uxt*^*F/Y*^ x Oz-Cre
	Observed
Female KO/+	9

### Germ cell specific deletion of *Uxt* results in a Sertoli cell-only phenotype

**Fig 1:**
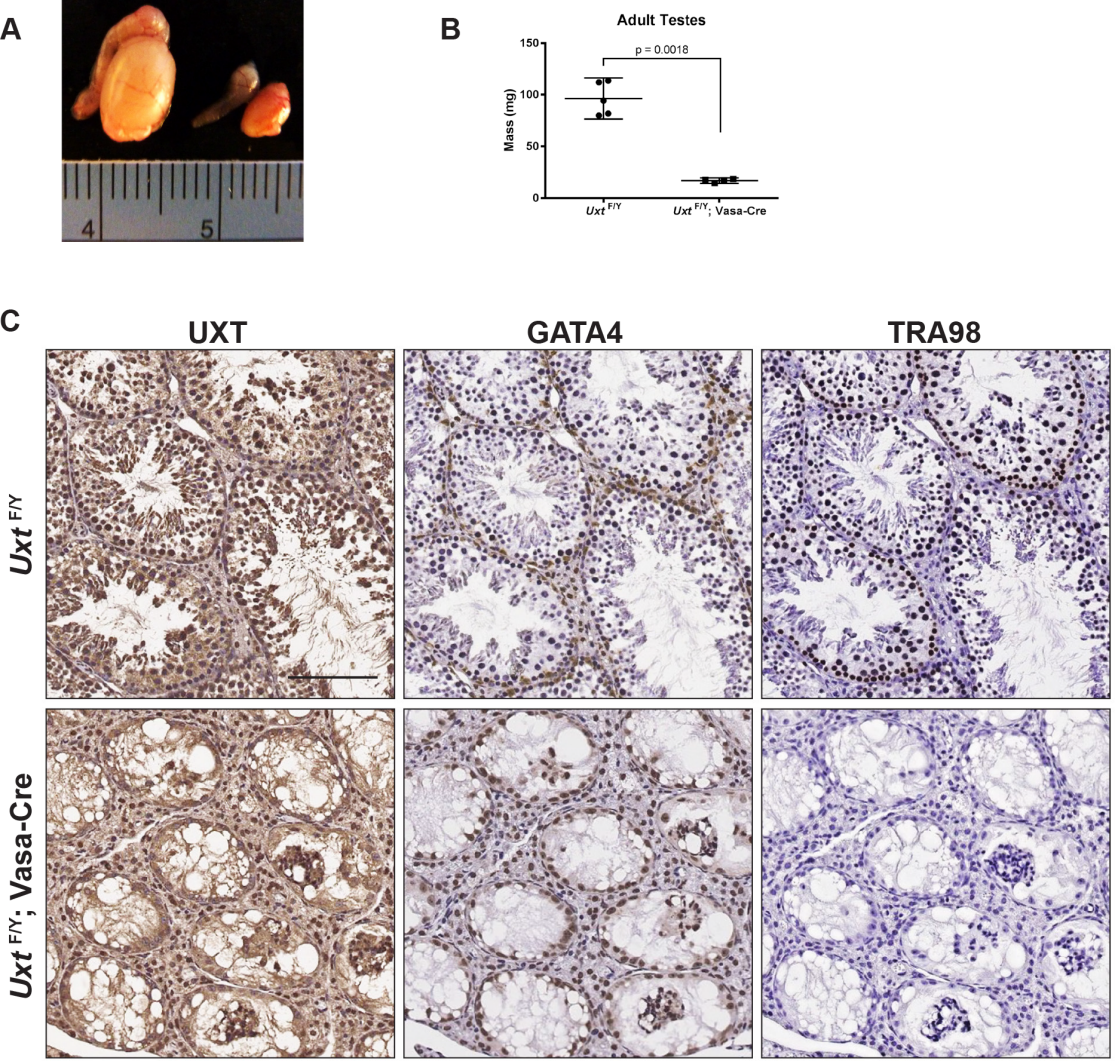
*Uxt* deletion results in complete germ cell loss in adult testes. (A) Gross anatomy of wild type (left) and *Uxt* ^*F/Y*^; Vasa-Cre (right) testes and epididymis. Numbers in ruler correspond to centimeters (cm). (B) Mass of *Uxt* ^*F/Y*^ littermate control versus *Uxt* ^*F/Y*^; Vasa-Cre testes in milligrams (mg) (n = 5; p = 0.0018), showing mean and 95% confidence interval (C.I.). Statistical significance was calculated using an unpaired Student’s t-test (p<0.0001). (C) Immunohistochemistry (IHC) for UXT, TRA98, and GATA4 in 5-month old *Uxt* ^*F/Y*^ and *Uxt* ^*F/Y*^; Vasa-Cre littermate testes sections. Positive staining is brown. Nuclei are labeled with hematoxylin counterstain in blue. Scale bars are 100 microns.

Given the embryonic lethality of UXT KO mice and the importance of URI in germ cells in lower organisms [15],we initiated studies of UXT in male germ cells. To induce germline-specific UXT deletion in the testes, we mated *Uxt* ^*F/F*^ females to Ddx4-Cre males (also known as Vasa-Cre). The Vasa-Cre transgene is expressed in gonocytes from embryonic day 15 to 18 and recombines with >97% efficiency [36]. At 5 months, *Uxt* ^*F/Y*^; Vasa-Cre testes (KO) were substantially smaller compared to *Uxt* ^F/Y^ littermate controls (WT), and weighed significantly less (Fig 1A and 1B). Immunohistochemical analysis was performed in 5 months old *Uxt* ^F/Y^ and *Uxt* ^*F/Y*^; Vasa-Cre testes for expression of UXT, GATA4, and TRA98 (Fig 1C). GATA4 and TRA98 was used as markers of Sertoli cells and germ cells [37–39], respectively. As expected, UXT is detected in all cell types in the *Uxt* ^F/Y^ testes, including germ cells at varying stages of spermatogenesis. In the *Uxt* ^*F/Y*^; Vasa-Cre testes, deletion of UXT resulted in a Sertoli cell-only phenotype [38], with complete loss of germ cells and absence of sperm in the epididymis (S2A Fig). UXT expression is however detected in the Sertoli and Leydig cells (Fig 1C). Examining *Uxt* ^*F/Y*^; Vasa-Cre testes where TRA98 positive germ cells are still present (S2B Fig), UXT expression is absent in germ cells as expected, while still present in the Sertoli cells.

### *Uxt* deletion in male germ cells abrogates meiosis and disrupts SSC homeostasis during the first wave of spermatogenesis

**Fig 2:**
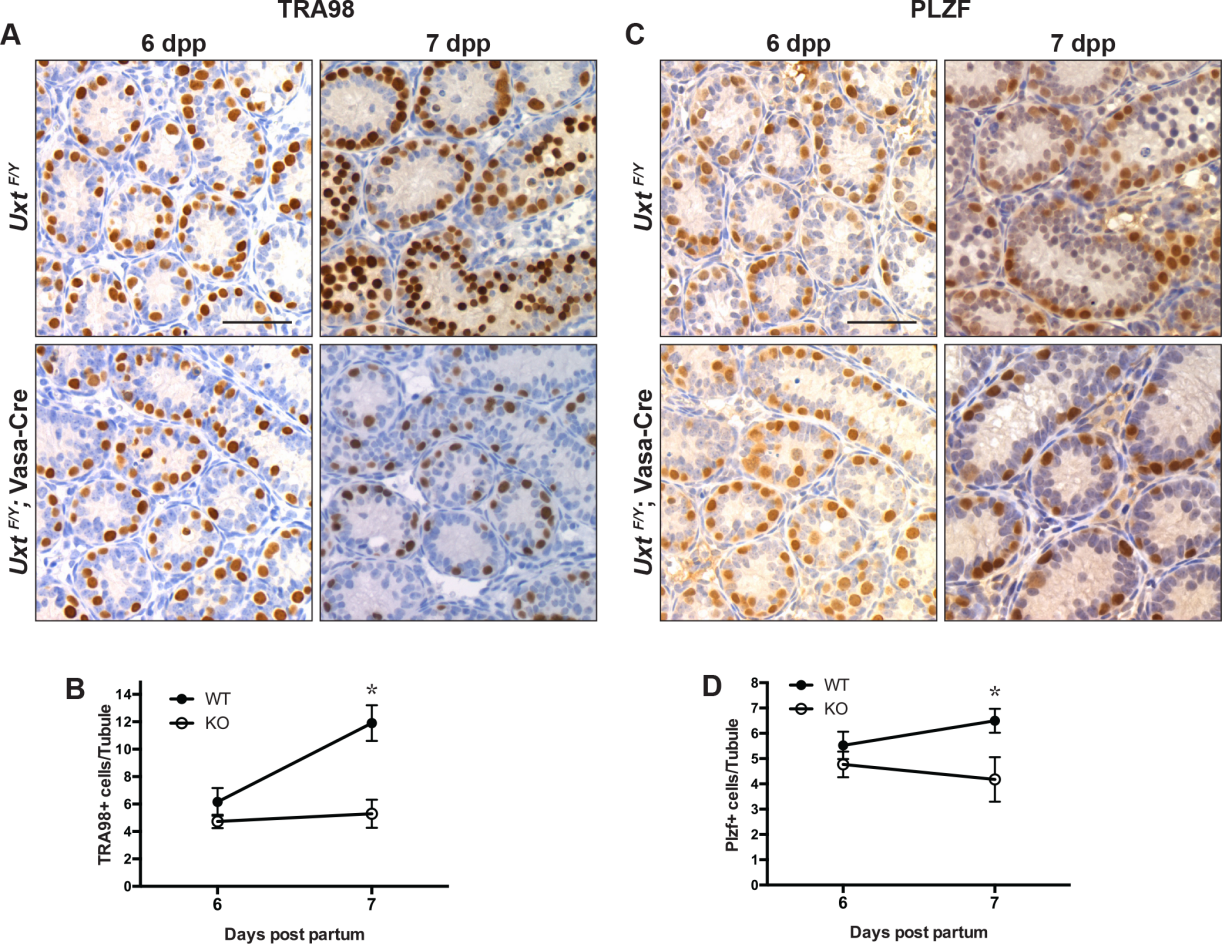
*Uxt* knockout suppresses germ cell expansion between 6 and 7 days post-partum (dpp). (A) IHC for TRA98 in representative tissue sections at 6dpp (left column) and 7dpp (right column) in *Uxt* ^*F/Y*^ and *Uxt* ^*F/Y*^; Vasa-Cre testes. (B) Mean number of TRA98-positive cells per tubule cross-section at 6dpp (n = 5; *p* = 0.64) and 7dpp (n = 5; *p = 0.024) in *Uxt* ^*F/Y*^ (WT) compared to *Uxt* ^*F/Y*^; Vasa-Cre (KO) testes. (C) IHC for PLZF in representative tissue sections at 6dpp (left column) and 7dpp (right column) in *Uxt* ^*F/Y*^ and *Uxt* ^*F/Y*^; Vasa-Cre testes. (D) Mean number of PLZF-positive cells per tubule cross-section at 6dpp (n = 7; *p* = 0.65) and 7dpp (n = 5;* *p* = 0.036) in *Uxt* ^*F/Y*^ compared to *Uxt* ^*F/Y*^; Vasa-Cre testes. The combined average number of positive cells per tubule from n mice is plotted. Error bars represent the standard error of the mean (S.E.M.). Unpaired Student’s t-tests were performed for all samples. Scale bars on the upper left micrographs are 50 microns.

We next sought to understand the developmental timing of germ cell loss by dissecting *Uxt* ^*F/Y*^; Vasa-Cre testes at various time points during the first wave of spermatogenesis by examining the expression of TRA98 and PLZF [9, 10]. We did not notice differences in testes morphology or numbers of germ cells through day 6, suggesting that typical numbers of gonocytes were formed and that spermatogenesis initiated normally. However, beginning at day 7, we observed a large difference in the number of TRA98-positive and PLZF-positive spermatogonia (undifferentiated spermatogonia) per tubule between wild type and UXT knockout testes (Fig 2). Furthermore, immunohistochemical staining for TRA98 revealed very few germ cells present at 14dpp and a Sertoli cell-only phenotype by 23dpp (S3 Fig), indicating that germ cell-specific *Uxt* deletion primarily affects the first wave of spermatogenesis. The depletion of TRA-positive cells in mutant animals is consistent with the fact that in UXT knockout testes, PLZF-positive and TRA98-positive spermatogonia do not increase in number between day 6 and day 7 as they do in the wild type. At day 7–8, numerous B spermatogonia and some preleptotene spermatocytes form a second layer of germ cells in the WT testes however, *Uxt* -null germ cells remain close to the basement membrane, suggesting their inability to proceed to a premeiotic stage (S4 Fig) [39]. Since all germ cells are completely lost during the first wave of spermatogenesis, germ cell-specific *Uxt* deletion must also necessarily prevent the juvenile SSC pool from self-renewing and establishing the second wave of spermatogenesis onward. There are four possible explanations for this phenotype. *Uxt* deletion either: 1) interferes with signaling pathways that induce differentiation and/or meiotic entry, 2) suppresses germ cell proliferation, 3) induces apoptosis as cells divide, enter meiosis, or lose “housekeeping” processes, or 4) a combination of these three.

### Analysis of apoptosis in UXT KO testes

With a previous role implicating UXT in mitochondrial aggregation that contributes to apoptosis, we investigated if *Uxt* knockout induced apoptosis [40, 41]. This could explain the large difference in germ cell number between wild type and knockout testes. To do this, we calculated the percentage of cleaved caspase 3-positive seminiferous tubules by staining 5, 6, and 7dpp tissue sections via immunohistochemistry (IHC) (S5 Fig). We found that the number of cleaved caspase-3-positive tubules did not differ between UXT knockout and wild type testes on 5 and 6dpp. However, at 7dpp, UXT knockout testes contained on average 1.5 times the number of cleaved caspase-3 positive tubules compared to wild type, with some knockout testes containing double the number of tubules containing apoptotic cells compared to wild type littermate controls (Table 3). While the overall frequency of cleaved caspase 3 is increased in the 7dpp knockout testes, we also observed that the average number of apoptotic cells per tubule does not differ between wild type and knockout testes, with between one and two cells per tubule staining positive. Altogether, the observed frequencies of apoptotic cells do not explain the approximately two-fold disparity in TRA98 positive germ cell number at 7dpp. However, apoptosis occurs rapidly, taking ten minutes on average [42] and making it difficult to catch *in vivo*. Further, germ cell loss may occur during a developmental window less than 24 hours, requiring a higher resolution analysis to capture the differences. It could also be that UXT knockout reduces germ cell proliferation and/or differentiation to a greater extent than its effect on cell death.

**Table 3:** Frequency of cleaved caspase 3-positive tubules.

5dpp		6dpp		7dpp	
WT (Frequency)	KO (Frequency)	WT (Frequency)	KO (Frequency)	WT (Frequency)	KO (Frequency)
11 / 121	15 / 117	21 / 96	20 / 92	10 / 102	25 / 99
0 / 93	0 / 113	3 / 99	1 / 53	12 / 135	14 / 137
1 / 104	1 / 90	0 / 100	7 / 95	15 / 95	37 / 110
3.77%	5.00%	10 / 117	15 / 123	16 / 113	12 / 97
*p = 0*.*45*		8.25%	11.85%	12 / 113	35 / 232
		*p = 0*.*10*		11.65%	18.22%
				*p <0*.*0001*	

Frequency of cleaved caspase-3-positive tubules/total number of counted tubules at days 5, 6, and 7dpp. Each row represents one biological replicate. To compare the proportion of apoptotic tubules between *Uxt* ^*F/Y*^ (WT) and *Uxt* ^*F/Y*^; Vasa-Cre (KO) testes, frequencies were pooled from each biological replicate and analyzed using the chi-squared test at each time point.

### *Uxt* deletion does not prevent spermatogonia cell cycle entry

**Fig 3:**
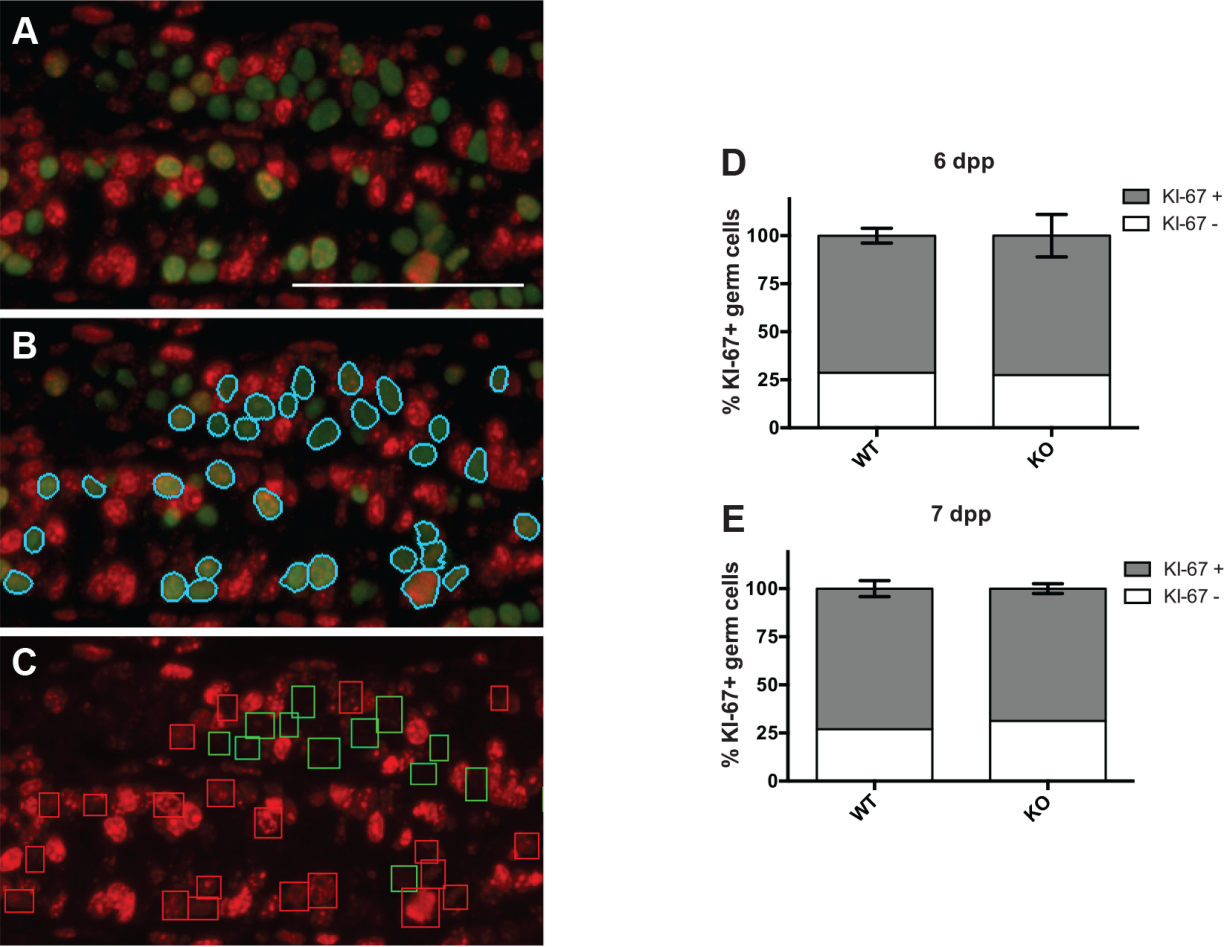
*Uxt* deletion does not affect cell cycle entry. (A) Immunofluorescence for TRA98 (green) and KI-67 (red) in a representative 6dpp *Uxt* ^*F/Y*^ testis section. (B) TRA98-positive nuclei after thresholding and segmentation are identified in cyan. (C) Identification of KI-67-positive and negative germ cell nuclei. Green boxes identify KI-67-negative germ cells. Red boxes identify KI-67-positive germ cells. Only the red channel is shown. (D) Percent KI-67 positive and negative germ cells at 6dpp (n = 3; chi-square test *p* = 0.91). (E) Percent KI-67 positive and negative germ cells at 7dpp (n = 4; chi-square test *p* = 0.18). Error bars represent S.E.M. Scale bar is 50 microns.

Another possible explanation for the germ cell phenotype is that *Uxt* deletion suppresses the proliferation of spermatogonia. To test this hypothesis, we performed co-immunofluorescence (IF) for TRA98 and MKI-67 (KI-67) and a machine learning algorithm to compare the percentage of germ cells entering the cell cycle between 6 and 7dpp in wild type and knockout testes (Fig 3A–3C). We found no difference in the percentage of KI-67-positive germ cells between wild type and UXT knockout at 6dpp (Fig 3D) or 7dpp (Fig 3E). Because KI-67 is only expressed upon cell cycle entry and not during G_0_ [43], it appears that *Uxt* deletion does not affect the proliferation or prevent cell cycle entry or exit from quiescence (Fig 2B and 2D, open circles).

### *Uxt* deletion results in misregulation of some differentiation pathways during the first wave of spermatogenesis

We next asked if *Uxt* deletion suppresses known signaling pathways implicated in germ cell differentiation and SSC self-renewal. To do this, we performed RNA-seq on whole 6dpp testes from *Uxt* ^*F/Y*^; Vasa-Cre and matched *Uxt* ^*F/Y*^ littermate controls from three separate litters. We chose 6dpp to capture differentially expressed genes foreshadowing germ cell depletion and to avoid false positives, since roughly equal numbers of germ cells are present between wild type and *Uxt* knockout testes (Fig 2B) at this stage. Seminiferous tubules of 5-day-old testes contain roughly 15% germ cells [44], and comprise an even smaller proportion of the entire testis when including Leydig cells. It is likely that a similar proportion exists at day 6, and thus proceeded to sequence 500 million reads (single-end, 50 base pair read length) to ensure adequate coverage of germ cell transcripts, which we considered to be greater than or equal to 10 million reads [45]. *Uxt* was downregulated 1.26-fold in the knockout testes with an adjusted p-value of 0.006 (Table 4A). Since Vasa-Cre recombines with high efficiency in germ cells, the low fold change may be attributed to the presence of other somatic testicular cells that express *Uxt* mRNA. *Id4* and *Gfrα*1 (germ cell specific), and *Gata4* (Sertoli and Leydig cell marker) are not differentially expressed (Table 4A and 4B) suggesting that there are similar numbers of germ cells and somatic cells at day 6 in wild type compared to UXT knockout testes.

**Table 4:** Differentially expressed genes in *Uxt*^*F/Y*^; Vasa-Cre testes.

**A: Controls**				**F: DNA methylation and piRNA processing**			
*Gene*	*Fold Change*	*P-value*	*FDR*	*Gene*	*Fold Change*	*P-value*	*FDR*
Uxt	-1.26	0.0068	0.1293	Dnmt3l	-2.56	9.55E-09	4.45E-06
Gapdh	1.08	0.2106	0.6935	Dnmt3b	-1.65	9.42E-11	9.14E-08
Gata4	1.14	0.0541	0.3792	Piwil2	-1.58	1.24E-08	5.56E-06
**B: SSC markers**				Sall3	-1.57	0.0029	0.0738
*Gene*	*Fold Change*	*P-value*	*FDR*	Tdrd1	-1.52	2.64E-07	6.09E-05
Nanog	1.17	0.2967	0.7967	Piwil4	-1.48	0.0024	0.0671
Gfra1	1.12	0.0814	0.4596	Tdrd9	-1.48	0.0001	0.0079
Id4	1.03	0.6633	1	Mael	-1.42	1.86E-07	4.71E-05
Neurog3	-1.63	5.40E-05	0.0041	Mov10l1	-1.4	7.74E-06	0.0009
Lin28a	-1.54	6.02E-07	0.0001	Tdrd5	-1.38	2.49E-05	0.0022
Zbtb16	-1.42	1.18E-05	0.0013	**G: Retinoic acid signaling**			
Pou5f1	-1.36	0.0072	0.1329	*Gene*	*Fold Change*	*P-value*	*FDR*
**C: Transcription factors and target genes**				Crabp1	-2.11	1.55E-16	9.01E-13
*Gene*	*Fold Change*	*P-value*	*FDR*	Rbp4	-1.75	1.08E-06	0.0002
Bcl6b	1.14	0.4089	0.4089	Rara	1.06	0.4354	0.9526
Etv5	1.13	0.4706	0.4706	Rarg	-1.55	1.29E-06	0.0002
Setdb2	-1.41	7.64E-05	0.0052	Rarb	-1.57	0.0037	0.0868
Sohlh2	-1.44	1.59E-07	4.17E-05	Sox3	-1.72	2.38E-10	2.18E-07
Dmrtb1	-3.67	4.44E-22	1.03E-17	Stra8	-1.72	1.60E-05	0.0016
**D: Meiotic scaffolds**				Kit	-1.44	5.75E-05	0.0043
*Gene*	*Fold Change*	*P-value*	*FDR*	Sohlh2	-1.44	1.59E-07	4.17E-05
Sycp2	-2.14	6.93E-06	0.0009	Sohlh1	-1.41	1.31E-06	0.0002
Mei4	-1.9	2.31E-07	5.48E-05	Sall4	-1.34	0.0004	0.0201
Smc1b	-1.87	6.16E-07	0.0001	**H: Wnt and Notch**			
Mei1	-1.83	3.37E-05	0.0028	*Gene*	*Fold Change*	*P-value*	*FDR*
Sycp1	-1.58	7.96E-06	0.001	Wnt7b	-2.17	1.12E-07	3.11E-05
**E: RNA binding**				Fzd10	-1.64	1.44E-05	0.0015
*Gene*	*Fold Change*	*P-value*	*FDR*	Wnt3	-1.44	0.0015	0.0474
Dazl	-1.75	3.23E-12	5.78E-09	Hey1	-1.55	2.32E-06	0.0004
Ddx4	-1.63	3.27E-09	2.00E-06				
Ddx25	-1.47	2.03E-07	5.07E-05				
Boll	-1.42	0.0009	0.0336				

We used *Uxt* as a benchmark to develop cutoffs to find differentially expressed genes in our dataset (p < 0.05; FDR 13%; counts >10). Using these cutoffs, we found 928 downregulated genes and 206 upregulated genes in *Uxt* knockout testes compared to littermate controls. Although the RNA-seq was performed on whole testes, our technical approach was sensitive enough to detect differences in several germ cell specific genes. Additionally, we validated changes in gene expression of representative, selected genes via qPCR (S6 Fig).

DAVID functional analysis [46] of the top 500 downregulated genes identified enrichment of GO biological process categories related to meiosis, cell cycle regulation, and gene transcription (Table 5). These results corroborate both our phenotypic analysis and previous studies demonstrating the importance of UXT in transcriptional regulation [16, 20]. Analysis of the top 200 upregulated genes revealed metabolic biological processes that are also differentially regulated by UXT (Table 6). A complete list of differentially expressed genes is provided, along with the top 20 up- and downregulated genes (S1 and S2 Tables).

**Table 5:** DAVID GO biological process: Top 500 downregulated genes in knockout.

*Term*	*Count*	*PValue*	*Fold Enrichment*
meiosis	27	2.37E-15	7.1
M phase of meiotic cell cycle	27	2.37E-15	7.1
meiotic cell cycle	27	4.36E-15	6.9
sexual reproduction	55	1.53E-14	3.3
gamete generation	50	2.95E-14	3.5
male gamete generation	43	5.48E-14	3.9
spermatogenesis	43	5.48E-14	3.9
reproductive process in a multicellular organism	55	1.73E-13	3.1
multicellular organism reproduction	55	1.73E-13	3.1
meiosis I	16	4.26E-12	10.5
cell cycle phase	46	5.26E-12	3.2
cell cycle process	50	2.01E-11	2.9
cell cycle	62	7.45E-10	2.3
regulation of transcription	153	1.55E-09	1.6
M phase	38	1.71E-09	3.1
reproductive cellular process	28	6.36E-09	3.7
synapsis	10	8.92E-09	13.6
chromosome organization involved in meiosis	10	8.92E-09	13.6
oogenesis	13	5.16E-08	7.7
germ cell development	20	5.41E-08	4.6
prophase	8	3.75E-07	14.2
meiotic prophase I	8	3.75E-07	14.2
reproductive developmental process	32	4.23E-07	2.8
female gamete generation	14	5.56E-07	5.8
male meiosis	9	5.81E-07	10.9
spermatid differentiation	14	2.33E-06	5.1
transcription	116	3.61E-06	1.5
spermatid development	13	6.68E-06	5.1
regulation of RNA metabolic process	98	2.06E-05	1.5
DNA methylation	8	3.67E-05	8.0

**Table 6:** DAVID GO biological process: Top 200 upregulated genes in knockout.

*Term*	*Count*	*PValue*	*Fold Enrichment*
cellular amino acid derivative metabolic process	9	5.84E-05	6.7
oxidation reduction	17	6.21E-04	2.6
biogenic amine metabolic process	6	9.71E-04	7.8
lipid catabolic process	7	0.0017	5.5
peptide metabolic process	4	0.0055	11.0
coenzyme metabolic process	6	0.0117	4.4
glutathione metabolic process	3	0.0215	13.1
negative regulation of epithelial cell proliferation	3	0.0269	11.6
cofactor metabolic process	6	0.0297	3.4
response to oxidative stress	4	0.0498	4.8

To uncover likely pathways explaining the *Uxt* -null phenotype, we focused on genes known to impact mammalian spermatogenesis. Since *Uxt* deletion leads to eventual SSC loss, we first compared expression levels of classical SSC markers and maintenance genes in wild type versus knockout testes. We found no changes in *Id4* or *Gfrα1* gene transcription, and no expression changes in the *Gfrα1* target genes *Etv5* and *Bcl6b* [47] (Table 4B and 4C). This suggests that there are equal numbers of A_s_ SSCs at 6dpp in wild type and knockout testes, and that *Uxt* deletion primarily affects later differentiation stages. However, several other transcription factors required for SSC homeostasis were downregulated in the absence of UXT, such as *Neurog3*, *Lin28a*, *Zbtb16*, and *Dnmt3l* (Table 4B and 4F). We observed reduced protein expression of ZBTB16 (PLZF) in the KO compared to WT testes starting at 7dpp (Fig 2D and S3A Fig), and possibly contributing to hindering the establishment of the stem cell pool maintaining the second and subsequent waves of spermatogenesis.

Consistent with the KI-67 analysis (Fig 3), we saw no change in transcript levels of the proliferative markers *Pcna* and *Mki67* (S2 Table). However, we observed a modest downregulation of *Ccnd1* and *Cdk6* (-1.37 fold each), which jointly regulate progression through G_1_ in mitotic cells [48]. Together these data suggest the importance of further cell cycle analysis.

### Initial differentiation of spermatogonia appears to be normal in *Uxt* KO testes

**Fig 4:**
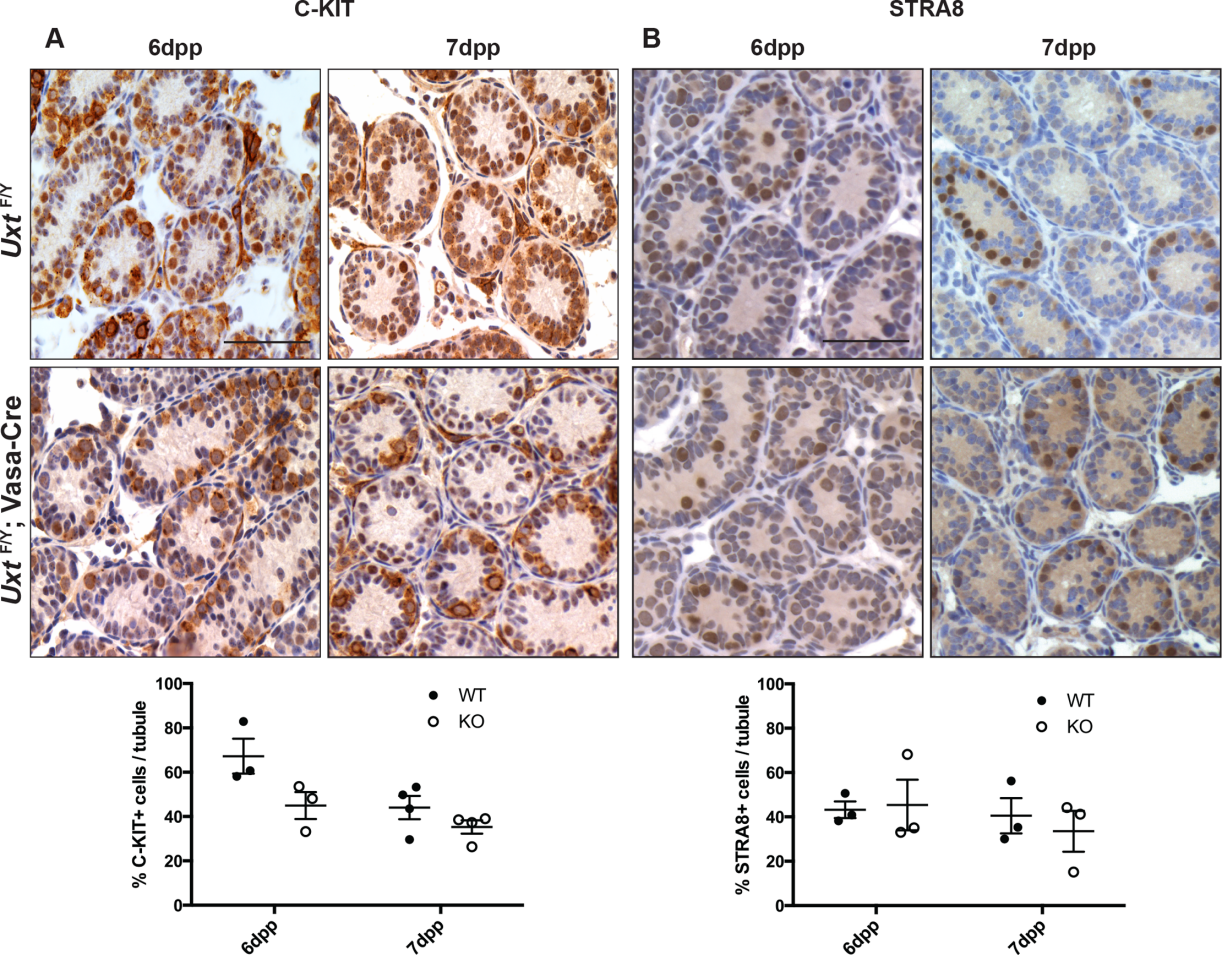
Analysis of spermatogonia differentiation in the *Uxt* knockout. IHC images for (A) C-KIT and (B) STRA8 at 6 and 7dpp in *Uxt* ^*F/Y*^ and *Uxt* ^*F/Y*^; Vasa-Cre testis sections. Quantification of C-KIT or STRA8 positive cells is shown below images. C-KIT levels in *Uxt* ^*F/Y*^ (WT) compared to *Uxt* ^*F/Y*^; Vasa-Cre (KO) testis sections (A) at 6dpp (n = 3; *p* = 0.10) and 7dpp (n = 4; *p* = 0.19) and STRA8 levels (B) in *Uxt* ^*F/Y*^ compared to *Uxt* ^*F/Y*^; Vasa-Cre testis sections at 6dpp (n = 3; *p* = 0.87) and 7dpp (n = 3; *p* = 0.60). Unpaired Student’s t tests were performed. Error bars represent S.E.M. Scale bars are 50 microns.

Retinoic acid signaling plays an important role in differentiation and meiosis of spermatocytes. Interestingly, several components of the RA signaling pathway were downregulated, including *Crabp1* [49], *Rbp4* [12], and the retinoic acid receptors *Rarβ* and *Rarγ* (Table 4G). We also observed a concomitant decrease in RA target gene transcript levels (Table 4G): *Sox3* [50], *Stra8* [51, 52], *C-kit* [53], *Sall4* [54], and *Sohlh1* [55, 56]. Given the decreased expression of several differentiation markers in our RNA-seq and qPCR data, we examined whether *Uxt* null spermatogonia undergo differentiation using antibodies against differentiation markers C-KIT and STRA8. By day 6, differentiated spermatogonia positive for both C-KIT and STRA8 are clearly detected in both KO and WT testes (Fig 4). At day 7, C-KIT positive spermatogonia are only detectable at the basal layer of the KO testes whereas in the WT testes the second layer of premeiotic cells starts forming (Fig 4A). Although the quantification of C-KIT and STRA8 protein expression did not reveal significant differences between WT and KO at the timepoints examined, the decrease in differentiation genes and lack of cells progressing to a second layer of premeiotic cells in the KO animals, suggest that initial differentiation of spermatogonia may be normal and that the major abnormalities are accumulating at the premeiotic stage. Genes related to meiosis are also downregulated. Of particular interest are *Dazl and Mael*, which affect spermatocytes in the leptotene and preleptotene stages, respectively. Deletion of *Mael* in the testes induces testis degeneration as early as 10dpp [57, 58]. Since UXT negative germ cells do not leave the basement membrane and thus do not develop past leptotene, the downregulation of *Mael* may be one of the contributing factors to this phenotype.

## Discussion

This is the first study examining the *in vivo* functional role of UXT in mammals; germ cell specific deletion of UXT results in a profound phenotype. Adult testes are significant smaller compared to WT animals and are devoid of sperm. The loss of germ cells begins at 6-7dpp in the KO animals and by 23dpp the testes are completely absent of germ cells, resulting in a Sertoli cell only phenotype. Although cell proliferation and apoptosis rates appear similar between WT and KO testes at 6 and 7 dpp, loss of UXT does have an apparent effect on the ability of cells to differentiate and enter meiosis. We found that UXT deletion affects transcriptional regulation of several pathways necessary for germ cell development that, when combined, result in a unique phenotype. To the best of our knowledge, this is the first phenotype exhibiting premeiotic arrest of spermatogenesis and loss of SSCs in a very narrow window during the first spermatogenic wave.

Following a prolonged period of quiescence, gonocytes resume proliferation starting at 3 dpp [59]. If UXT depletion caused general mitotic or housekeeping defects, we would expect significantly fewer germ cells starting at day 3–4 as opposed to 6–7 dpp compared to wild type. Given our gene expression data, we believe it is more likely that germ cells are arrested before they enter prophase of meiosis I, and that UXT has a function promoting the activation of transcriptional programs required for spermatogenesis. The fact that specific pathways are downregulated upon *Uxt* deletion is consistent with previously reported roles for UXT as a co-factor for various transcription factors [19–22], and may indicate that UXT has more specific functions in germ cells as opposed to a general housekeeping role in gene transcription.

Expression of several important germ cell specific genes was downregulated in the absence of UXT including genes regulating spermatogonia differentiation and meiosis. Retinoic acid signaling is of interest since many mouse models with impaired RA metabolism retain only A_undiff_ spermatogonia or low numbers of A_diff_ spermatocytes in adulthood, however in contrast we observe complete germ cell loss by 23dpp. To observe complete germ cell loss in adulthood, *Uxt* deletion must also hinder SSC self-renewal contributing to the second wave of spermatogenesis. Indeed, our data showed PLZF is decreased in our KO animals which in addition to reduced expression of *Lin28a*,*Dnmt3l*, and/or *Ngn3* could be responsible for this phenomenon. Loss of UXT seems to affect genes involved in the transition from A_al_ to A_1_ (*Dnmt3b*) and A_4_ to In/B (*Dmrtb1* (*Dmrt6*)), and A_1-4_ spermatogonia (*C-kit*, *Stra8*, *Sohlhl 1* and *2*). Future studies are warranted to elucidate the mechanism by which UXT affects differentiation and meiosis.

ChEA2 analysis was employed to find putative transcription factors involved in the regulation of differentially expressed gene in the UXT knockout mouse [60]. We found highly significant enrichment for SUZ12, a polycomb group protein [61], and SETDB1 (S3 Table). Polycomb proteins are important transcriptional regulators of stem cell fate and are important for terminal differentiation of male germ cells in *Drosophila* [62, 63]. Their functions in mammalian germ cells are poorly understood. *Ngn3* and *Plzf* contained putative SUZ12 binding sites as well. A recent study indicates that UXT directly interacts with SUZ12 and EZH1 to help load RNA Pol II to target genes in 293FRT cells [21]. It is possible that UXT could be affecting transcriptional regulation in germ cells by interacting with polycomb transcription factors (TFS), a topic of future investigation.

One potential binding partner of UXT in spermatogonia, is URI-1. Germline deletion of URI-1 in *C*. *elegans* and *Drosophila* results in DNA damage, germ cell loss, and subfertility in the male germline [14, 15]. Our lab has shown that UXT knockdown reduces URI-1 protein stability in prostate cancer cells [16]. If the function of URI-1 is conserved in mammals, it is possible that *in vivo* deletion of UXT would result in URI-1 protein destabilization and thus negatively impact germ cell homeostasis in the testes.

Here we demonstrated a previously unexplored requirement for UXT in spermatogenesis. A number of pathways essential for post-natal germ cell development are misregulated upon *Uxt* deletion, consistent with its previously defined role as a transcriptional co-factor. *Uxt* deletion does not seem to impair a single signaling pathway. Rather, the downregulation of multiple pathways produces a unique phenotype affecting both differentiation of premeiotic cells during the first wave of spermatogenesis and spermatogonia stem cell fate. Future studies will confirm the specific role of UXT in regulation of genes and pathways identified in the current study as well as identify UXT-interacting proteins. Together these studies may uncover novel mechanisms regulating specific developmental programs during a critical window of spermatogenesis, and dissect the mechanism by which UXT regulates meiotic entry and maintenance of stem cells.

## Supporting information

S1 File Supporting information: Materials and methods. (DOCX)

S1 Fig Conditional deletion of the *Uxt* allele. (A) Schematic representation of the wild type *Uxt* locus (*Uxt* ^*WT*^), targeting cassette, floxed, and knockout alleles. Starting at exon 2, coding exons are depicted by black vertical boxes numbered in white. Non-coding exons are depicted with white boxes. The locations of the endogenous start (ATG) and stop (TGA) codons at the wild type *Uxt* locus are labeled with black balls and sticks (*Uxt* ^*WT*^). A neomycin-resistance cassette (PGK-Neo-pA) introducing a novel BamHI restriction site (green) and flanked by FRT sites (*Uxt* ^*Neo;Flox*^) was engineered into the *Uxt* locus for selection purposes. This allele also contains loxP sites (red horizontal boxes) flanking the FRT sites and exon 3 of *Uxt*, and a novel BamHI restriction site (labeled in green). *Uxt* ^*Neo;Flox*^ mice were subsequently mated to a FLPase deleter strain, resulting in excision of the neomycin resistance cassette while leaving the loxP sites and the novel BamHI restriction site intact (*Uxt* ^*Flox*^). The approximate location of the Southern blot probe is also depicted (enP). CRE-mediated recombination results in removal of exon 3 and a 1-base pair frameshift affecting exons 4 and 5 (red vertical boxes), and also introducing a novel STOP codon in exon 5 (red ball and stick, TGA). This produces the knockout allele (*Uxt* ^*KO*^). (B) Example Southern blot showing hybridization of the enP probe to the *Uxt* ^*WT*^ and *Uxt* ^*Flox*^ alleles after digesting genomic DNA with BamHI. Note that females are heterozygous (last two lanes) and males are hemizygous because *Uxt* is located on the X-chromosome. (C) PCR genotyping of genomic DNA using primers recognizing each *Uxt* allele. (D) 15P-1 cells (mouse Sertoli cell line) transfected with siRNAs against control or mouse UXT. Immunoblot using affinity purified mouse UXT antibody and ERK1 (loading control) as indicated.
(TIF)

S2 Fig Absence of sperm in the epididymis and UXT expression as a result of *Uxt* deletion. (A) Periodic acid-Schiff (PAS) staining of *Uxt* ^*F/Y*^ and *Uxt* ^*F/Y*^; Vasa-Cre testis and epididymis sections. Scale bar is 100 microns. (B) Testes from *Uxt* ^*F/Y*^ and *Uxt* ^*F/Y*^; Vasa-Cre mice at 7dpp are stained using antibodies against UXT or TRA98. Arrows indicate the absence of UXT expression in TRA98 positive germ cells in the KO testes.
(TIF)

S3 Fig Germ cell-specific *Uxt* deletion results in a Sertoli cell-only phenotype by 23dpp. (A) IHC on serial sections for TRA98 (left column), PLZF (middle column), and GATA4 (right column) in 14dpp *Uxt* ^*F/Y*^ and *Uxt* ^*F/Y*^; Vasa-Cre littermates. (B) IHC on serial sections for TRA98 (left column) and GATA4 (right column) in 23dpp *Uxt* ^*F/Y*^ and *Uxt* ^*F/Y*^; Vasa-Cre littermates. Scale bars are 50 microns.
(TIF)

S4 Fig *Uxt* -null germ cells remain close to the basement membrane. (A) IHC on serial sections for TRA98 (left column) and PLZF (right column) on 8dpp *Uxt* ^*F/Y*^ and *Uxt* ^*F/Y*^; Vasa-Cre littermate testis. (B) IHC for TRA98 (left column) and PLZF (right column) in 10dpp in *Uxt* ^*F/Y*^ and *Uxt* ^*F/Y*^; Vasa-Cre littermate testis sections. Arrows point to germ cells (TRA98) and undifferentiated spermatogonia (PLZF). Scale bars are 50 microns.
(TIF)

S5 Fig Analysis of apoptosis in *Uxt* knockout at 7dpp. IHC for cleaved caspase-3 on 5 (left column), 6 (middle column), and 7dpp (right column) testis tissue sections from *Uxt*^*F/Y*^ and *Uxt*^*F/Y*^; Vasa-Cre littermates. Scale bars are 50 microns.
(TIF)

S6 Fig qPCR Validation of *Uxt*^F/Y^ Vasa-Cre RNA-seq. RNA was collected from 6dpp old *Uxt* ^F/Y^; Vasa-Cre and *Uxt* ^F/Y^ littermate controls and qPCR was conducted on selected genes. Gene expression is shown relative to GAPDH.
(TIF)

S1 Table **Top 20 differentially expressed (A) upregulated and (B) downregulated genes between *Uxt*** ^***F/Y***^**; Vasa-Cre and *Uxt*** ^***F/Y***^ **littermate controls from the RNA-seq dataset.** Gene symbols, gene name, fold change, adjusted p-value, and false discovery rate are shown.
(XLSX)

S2 Table Excel spreadsheet of RNA-seq data. Filters include logFC > 0 and Counts > 10. Three littermate pairs were sequenced: WT1/KO2; WT3/KO4; and WT5/KO6. Column values in each WT/KO category are counts.
(XLSX)

S3 Table **ChEA2 analysis of the top 20 up (A) and downregulated (B) genes upon *Uxt* knockout.** Overlap indicates the number of genes in the dataset with binding sites for that transcription factor (TF) compared to the total number of binding sites found for that TF in the database. Multiple ChIP experiments have been carried out for that particular TF (“Term” column) if the term is repeated in the list.
(XLSX)
