# Supplementary material for: UXT is required for spermatogenesis in mice
Source: PLoS One. 2018 Apr 12;13(4):e0195747. doi: 10.1371/journal.pone.0195747 (PMC5896988; doi:10.1371/journal.pone.0195747)
Supplement: S1 File — (DOCX) [file pone.0195747.s001.docx]

**S1 File. Supporting Information: Materials and Methods**

**Southern blotting**

10-20μg of genomic DNA (gDNA) from mouse tail biopsies were digested with BamHI and separated on a 0.8% agarose gel. Depurination of gDNA was carried out by soaking the gel in 0.25M HCl, followed by neutralization and denaturation of DNA in 1.5M NaCl plus 0.5M NaOH. The gDNA was then transferred onto a Duralon-UV nylon membrane via capillary action overnight, and crosslinked to the membrane using a Stratalinker UV-crosslinker (Stratagene). Next, the membrane was pre-hybridized in buffer (0.25M NaP, 7% SDS, 15% formamide, 1mM EDTA, 5% BSA w/v) for 4-6 hours at 60°C. A 517 base pair probe (enP) corresponding to the intron between exons 2 and 3 of *Uxt* was generated from an *in vitro* transcription reaction incorporating ^32^P dCTP nucleotides (Rediprime II DNA labeling system, GE Healthcare Life Sciences). Unincorporated nucleotides were removed using a NucAway Spin Column (Thermo Fisher Scientific). We used probes with a specific activity of at least 10^8^ cpm/μg. To detect genomic DNA fragments, the probe was boiled, added to fresh hybridization buffer (composition described above), and hybridized to the membrane in a rolling oven overnight at 60°C. The membrane was washed several times in low-stringency buffer (0.15M NaP, 0.1% SDS) and visualized on autoradiography film (Denville Scientific).

## Genotyping PCR

Genomic DNA was isolated using the KAPA Express Extract DNA Extraction Kit (KAPA Biosystems) according to the manufacturer’s instructions. PCR was performed using Promega GoTaq with 4mM MgCl_2_ using the following *Uxt* primers: 5’-CCCAGTGCCTGAAATACACTAAAGACTC-3’ and 5’-GGTAGGTGTCAGCGGGCAATTTTA-3’. gDNA was amplified for 30 cycles of 95°C for 30 seconds, 63°C for 30 seconds, and 72°C for 90 seconds, followed by a final 10-minute extension at 72°C. Bands were separated on a 1.2% agarose gel and visualized using ethidium bromide.

**PAS staining**

For Periodic Acid Schiff (PAS) staining, testes were dissected and fixed in Bouin’s solution (Sigma-Aldrich) overnight at 4°C without nutation, then dehydrated for three changes each for 20 minutes of 50% and 70% ethanol. Testes were then incubated at room temperature in 70% ethanol saturated with lithium chloride until tissue turned white to neutralize residual picric acid. After fixation, tissues were dehydrated in 50%, 70%, 95%, and 100% ethanol for three changes of 20 minutes each, followed by clearing with two 10 minute changes of xylenes, and overnight paraffin infiltration at 60°C.

5μm sections from Bouin’s fixed, paraffin-embedded testes were deparaffinized and re-hydrated as described above. Sections were then stained for 15 minutes in a fresh solution of 0.5% aqueous periodic acid (Fisher), rinsed in distilled water, and stained for 15 minutes in Schiff’s reagent (Sigma-Aldrich). Nuclei were counterstained with Gill’s III hematoxylin (Richard Allan), followed by differentiation in 70% ethanol/acetic acid and bluing in a ~3% ammonia solution. Slides were then dehydrated through a graded series of ethanol baths and xylene, then mounted with Permount (Fisher) and coverslipped.
