# Supplementary figures and images for: UXT is required for spermatogenesis in mice
Source: PLoS One. 2018 Apr 12;13(4):e0195747. doi: 10.1371/journal.pone.0195747 (PMC5896988; doi:10.1371/journal.pone.0195747)

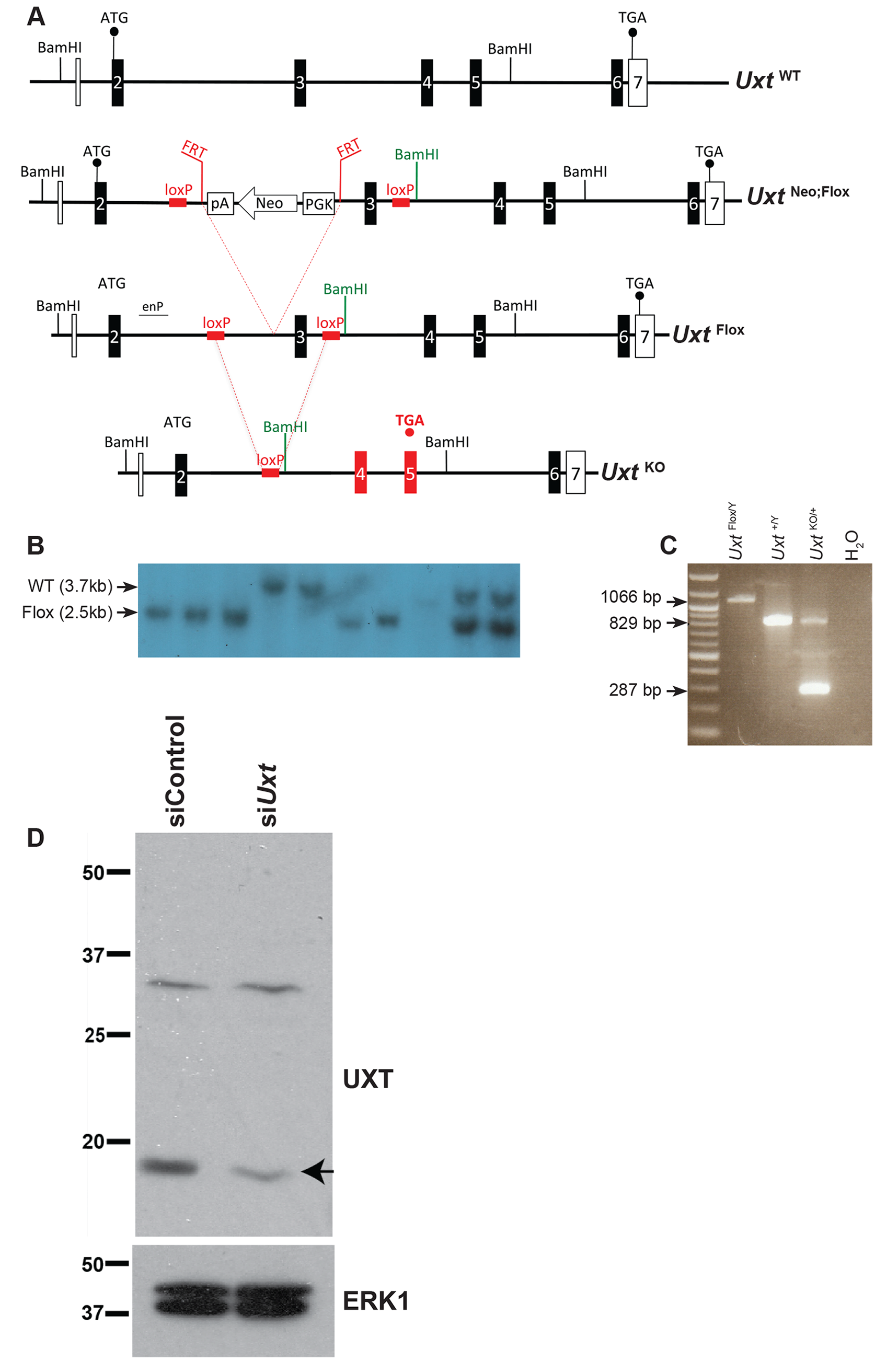

Supplement: S1 Fig — (A) Schematic representation of the wild type Uxt locus (Uxt WT), targeting cassette, floxed, and knockout alleles. Starting at exon 2, coding exons are depicted by black vertical boxes numbered in white. Non-coding exons are depicted with white boxes. The locations of the endogenous start (ATG) and stop (TGA) codons at the wild type Uxt locus are labeled with black balls and sticks (Uxt WT). A neomycin-resistance cassette (PGK-Neo-pA) introducing a novel BamHI restriction site (green) and flanked by FRT sites (Uxt Neo;Flox) was engineered into the Uxt locus for selection purposes. This allele also contains loxP sites (red horizontal boxes) flanking the FRT sites and exon 3 of Uxt, and a novel BamHI restriction site (labeled in green). Uxt Neo;Flox mice were subsequently mated to a FLPase deleter strain, resulting in excision of the neomycin resistance cassette while leaving the loxP sites and the novel BamHI restriction site intact (Uxt Flox). The approximate location of the Southern blot probe is also depicted (enP). CRE-mediated recombination results in removal of exon 3 and a 1-base pair frameshift affecting exons 4 and 5 (red vertical boxes), and also introducing a novel STOP codon in exon 5 (red ball and stick, TGA). This produces the knockout allele (Uxt KO). (B) Example Southern blot showing hybridization of the enP probe to the Uxt WT and Uxt Flox alleles after digesting genomic DNA with BamHI. Note that females are heterozygous (last two lanes) and males are hemizygous because Uxt is located on the X-chromosome. (C) PCR genotyping of genomic DNA using primers recognizing each Uxt allele. (D) 15P-1 cells (mouse Sertoli cell line) transfected with siRNAs against control or mouse UXT. Immunoblot using affinity purified mouse UXT antibody and ERK1 (loading control) as indicated. (TIF) [file pone.0195747.s002.tif]

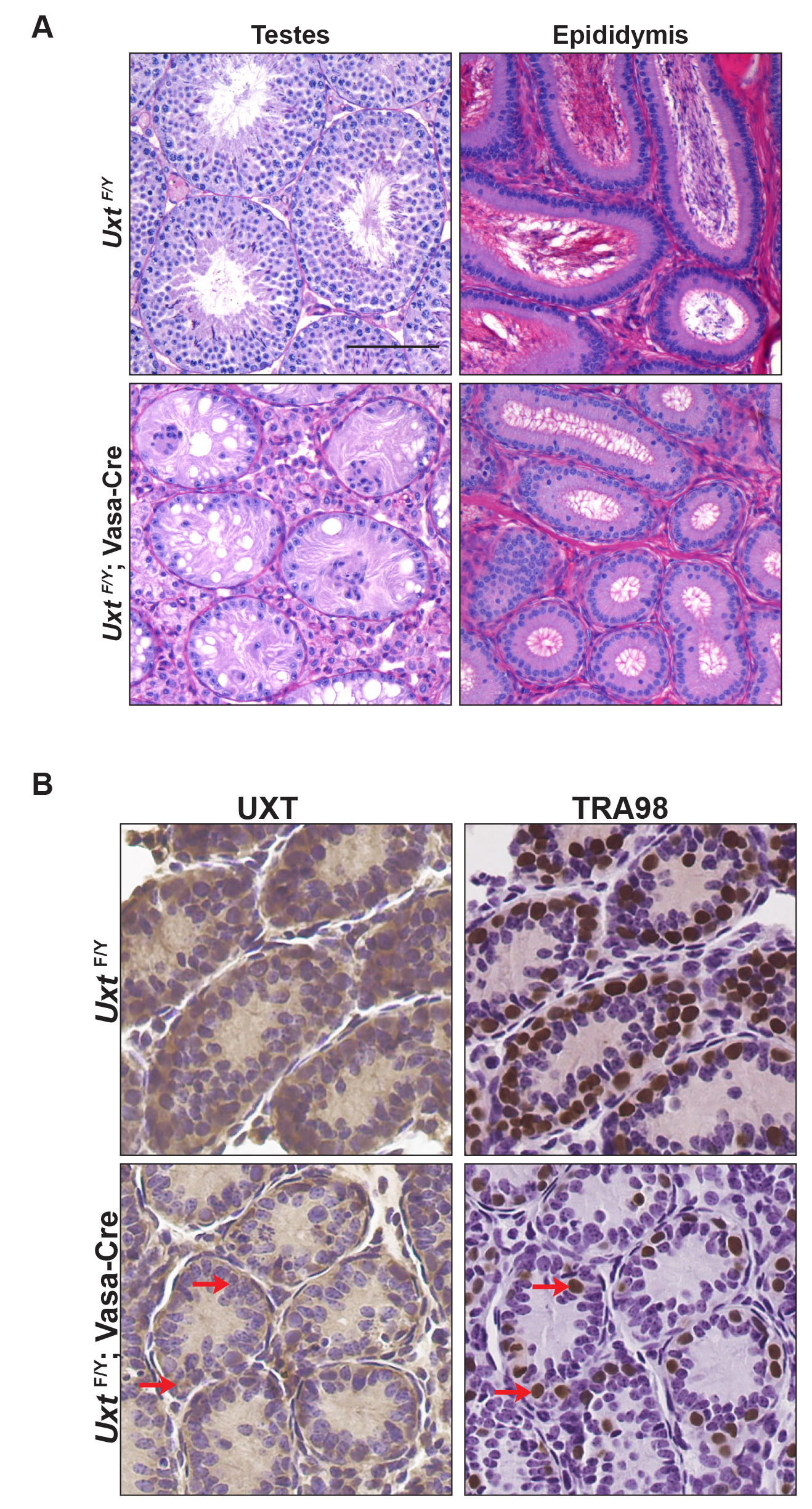

Supplement: S2 Fig — (A) Periodic acid-Schiff (PAS) staining of Uxt F/Y and Uxt F/Y; Vasa-Cre testis and epididymis sections. Scale bar is 100 microns. (B) Testes from Uxt F/Y and Uxt F/Y; Vasa-Cre mice at 7dpp are stained using antibodies against UXT or TRA98. Arrows indicate the absence of UXT expression in TRA98 positive germ cells in the KO testes. (TIF) [file pone.0195747.s003.tif]

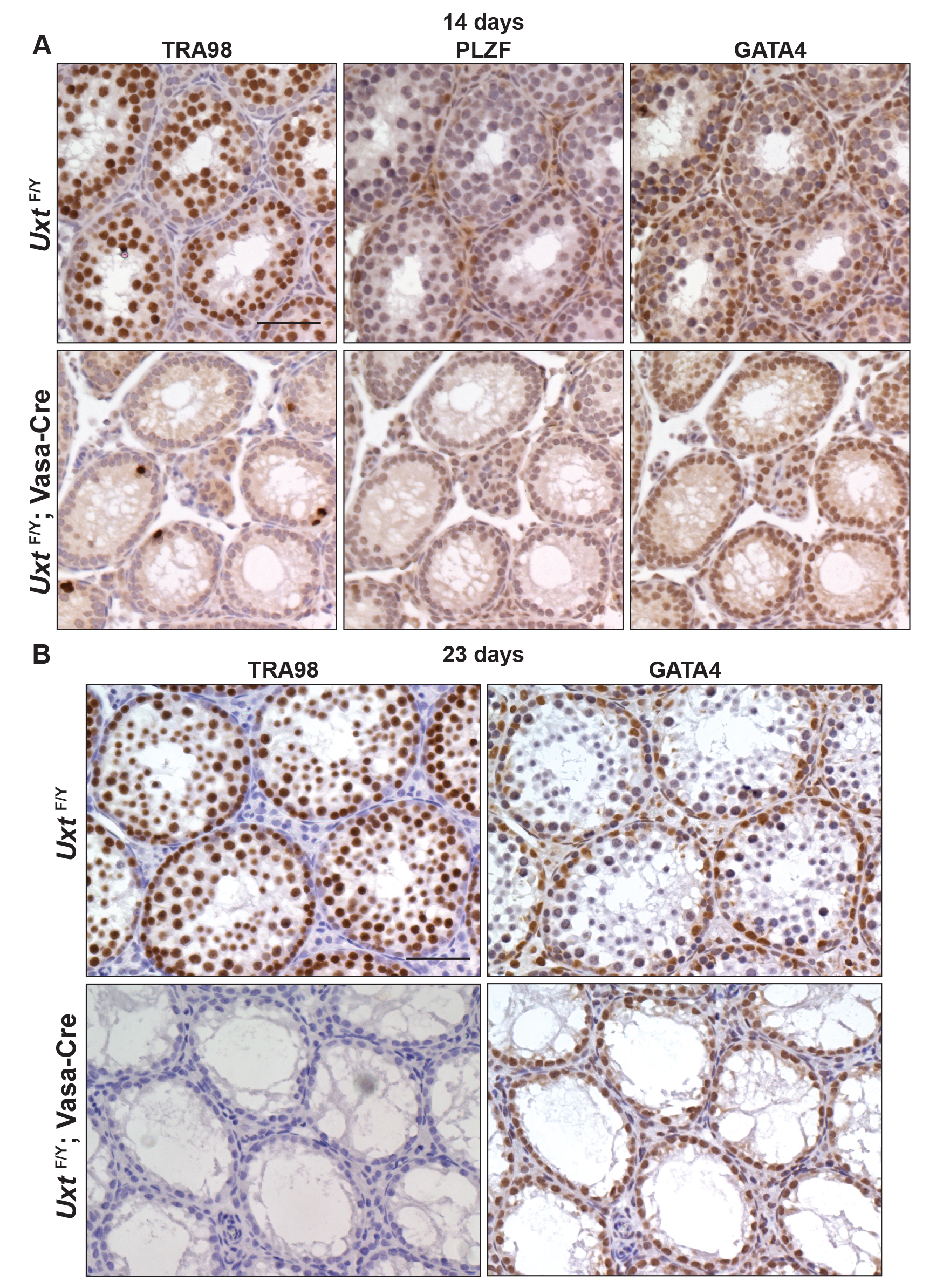

Supplement: S3 Fig — (A) IHC on serial sections for TRA98 (left column), PLZF (middle column), and GATA4 (right column) in 14dpp Uxt F/Y and Uxt F/Y; Vasa-Cre littermates. (B) IHC on serial sections for TRA98 (left column) and GATA4 (right column) in 23dpp Uxt F/Y and Uxt F/Y; Vasa-Cre littermates. Scale bars are 50 microns. (TIF) [file pone.0195747.s004.tif]

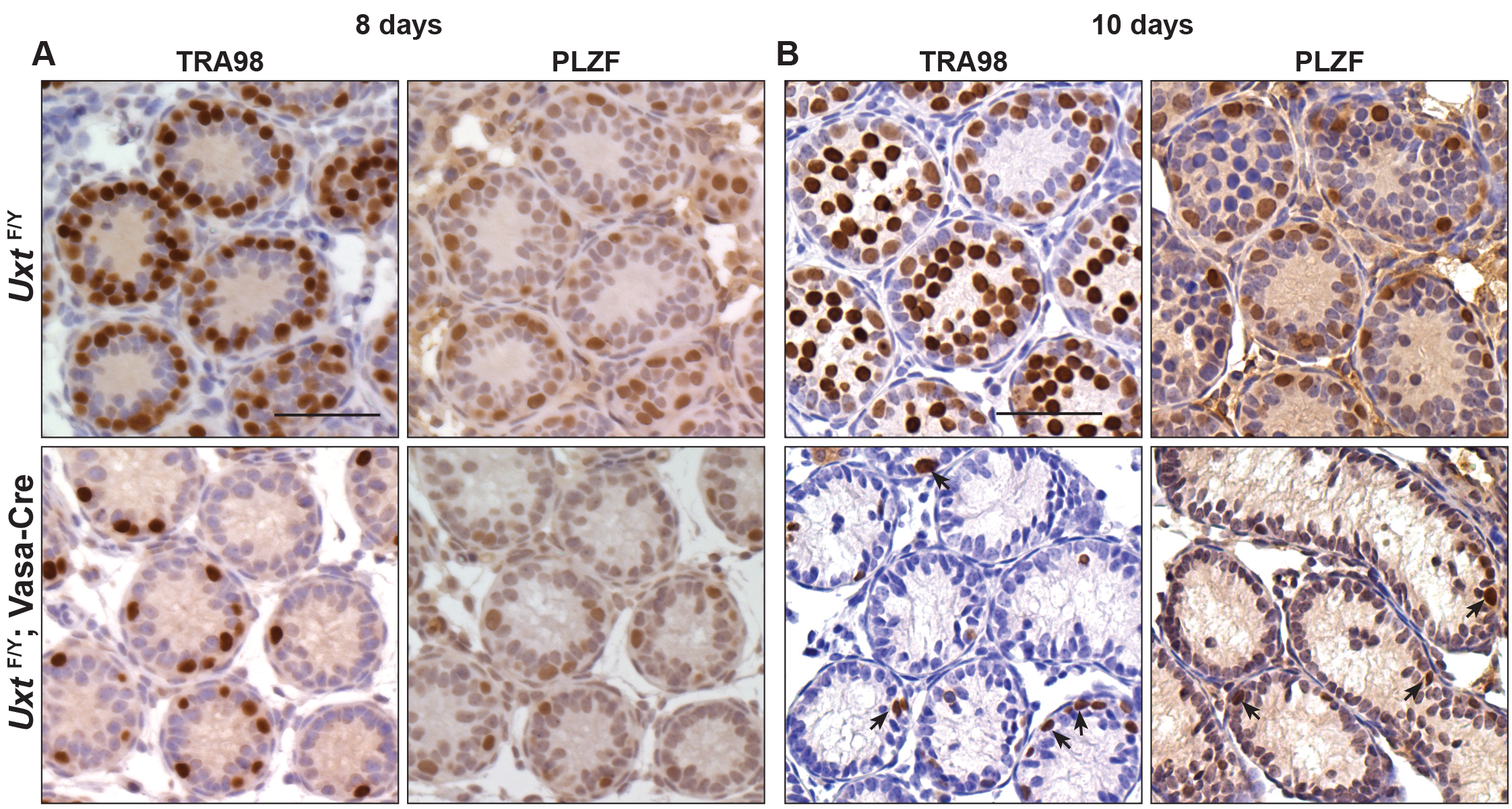

Supplement: S4 Fig — (A) IHC on serial sections for TRA98 (left column) and PLZF (right column) on 8dpp Uxt F/Y and Uxt F/Y; Vasa-Cre littermate testis. (B) IHC for TRA98 (left column) and PLZF (right column) in 10dpp in Uxt F/Y and Uxt F/Y; Vasa-Cre littermate testis sections. Arrows point to germ cells (TRA98) and undifferentiated spermatogonia (PLZF). Scale bars are 50 microns. (TIF) [file pone.0195747.s005.tif]

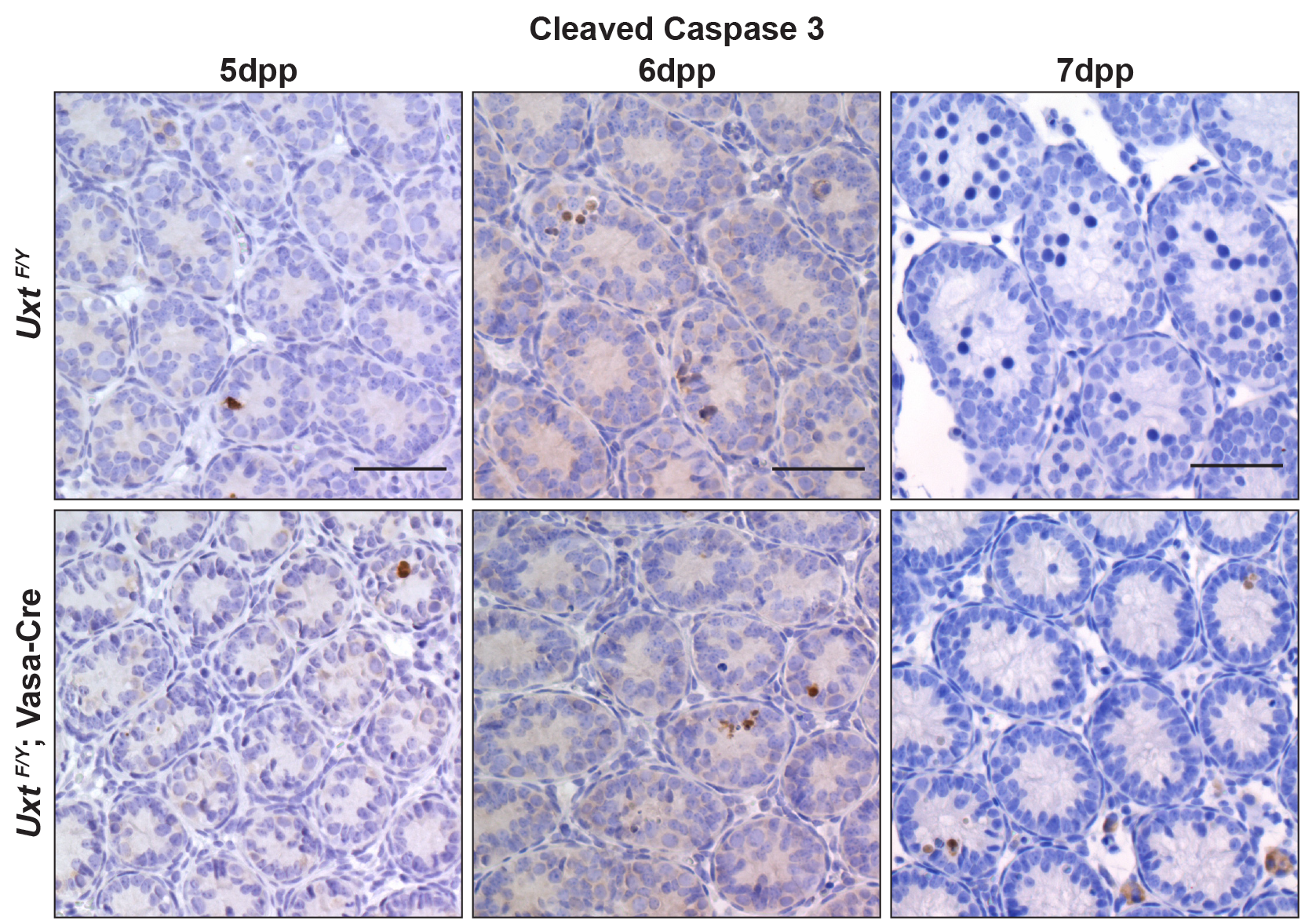

Supplement: S5 Fig — IHC for cleaved caspase-3 on 5 (left column), 6 (middle column), and 7dpp (right column) testis tissue sections from UxtF/Y and UxtF/Y; Vasa-Cre littermates. Scale bars are 50 microns. (TIF) [file pone.0195747.s006.tif]

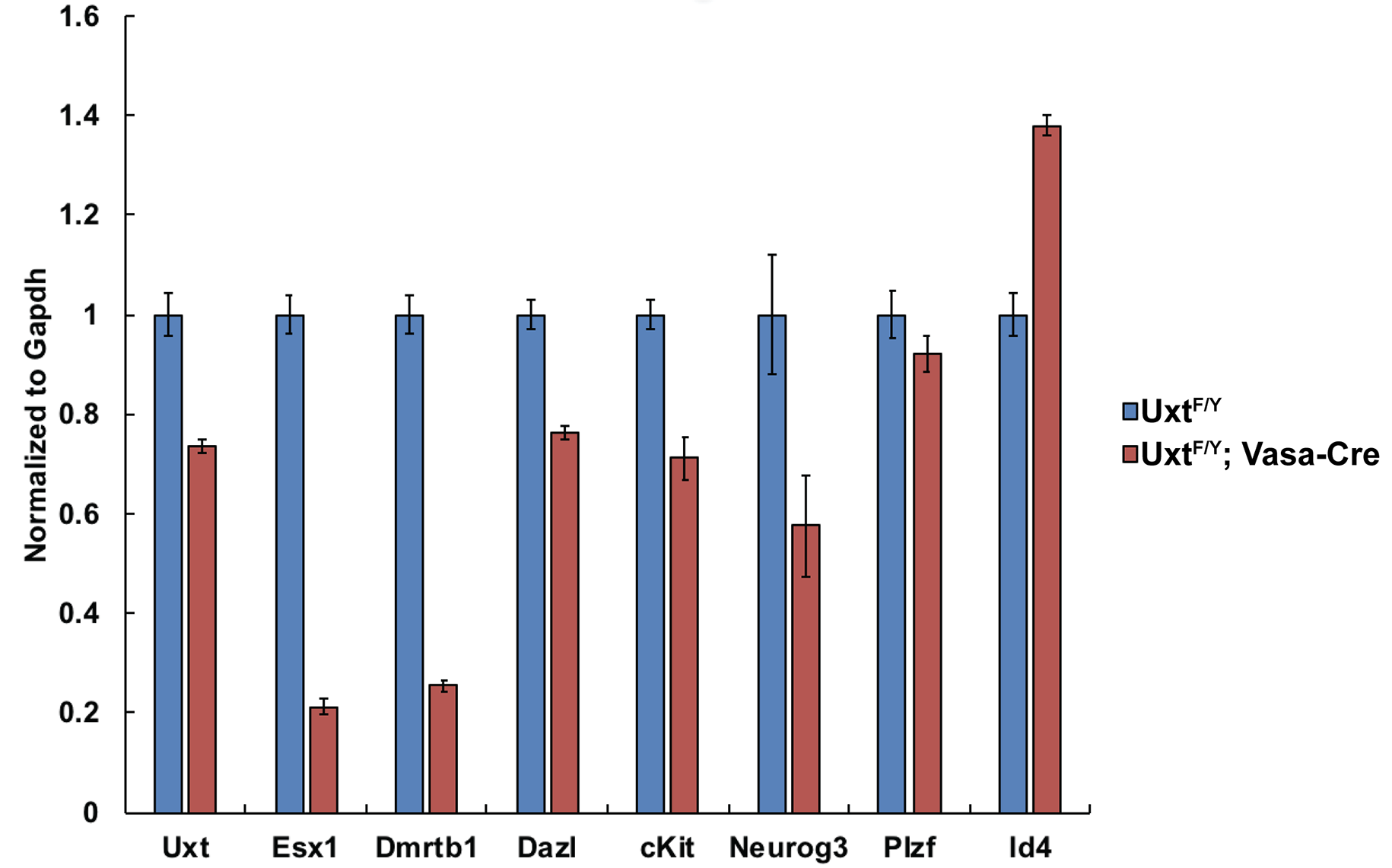

Supplement: S6 Fig — RNA was collected from 6dpp old Uxt F/Y; Vasa-Cre and Uxt F/Y littermate controls and qPCR was conducted on selected genes. Gene expression is shown relative to GAPDH. (TIF) [file pone.0195747.s007.tif]
